# Absence of TCL1A expression is a useful diagnostic feature in splenic marginal zone lymphoma

**DOI:** 10.1007/s00428-012-1322-z

**Published:** 2012-10-14

**Authors:** Enrico Munari, Marianna Rinaldi, Achille Ambrosetti, Massimiliano Bonifacio, Angela Bonalumi, Marco Chilosi, Alberto Zamò

**Affiliations:** 1 Department of Pathology and Diagnostics, Section of Anatomic Pathology, University of Verona, P.le Scuro 10, 37134 Verona, Italy; 2 Department of Medicine, Section of Haematology, University of Verona, P.le Scuro 10, 37134 Verona, Italy

**Keywords:** Splenic marginal zone lymphoma, TCL1A, Immunohistochemistry, Differential diagnosis

## Abstract

Splenic marginal zone lymphoma (SMZL) is a low-grade lymphoma showing a rather nonspecific immunophenotype. Gene expression profiling studies suggested that TCL1A could be a marker of SMZL, but reported data are conflicting. We evaluated TCL1A expression in a series of spleen and bone marrow samples involved by SMZL and correlated the findings with other immunophenotypical, morphological, and clinical data. In addition, we evaluated the expression of TCL1A in a series of spleens and lymph nodes involved by lymphomas that might mimic SMZL (13 nodal marginal zone lymphomas (NMZL), 39 follicular lymphomas (FL), 30 B-cell chronic lymphocytic leukemias (B-CLL), 31 mantle cell lymphomas (MCL), 1 lymphoplasmacytic lymphoma) and 15 bone marrow specimens involving hairy cell leukemia (HCL). TCL1A staining was negative in 24/31 cases of SMZL (77 %); 27/31 MCL and all B-CLL were positive for TCL1A; 32/34 cases of nodal FL (96 %) and all five splenic FL were positive for TCL1A, although at a lower intensity. Eight of 13 NMZL were positive for TCL1A, often showing a heterogeneous staining pattern. All HCL samples were strongly positive for TCL1A. No correlation was found between the pattern of splenic infiltration, TCL1A expression, and the clinical course. TCL1A-positive SMZL showed a higher rate of DBA44 staining compared to the negative ones, and this difference was statistically significant (Fisher test, single-tailed, *p* = 0.0397). Our data support the use of TCL1A in the panel of diagnostic markers used in the differential diagnosis of splenic low-grade B-cell lymphoma; a possible prognostic value, however, needs a larger series to be established.

## Introduction

Splenic B-cell marginal zone lymphoma is a rare entity, accounting for less than 2 % of all lymphoid malignancies; it shows an indolent clinical course, although 10 % of cases progress to diffuse large B cell lymphoma [1]. The diagnosis of SMZL relies on a combination of histopathological analysis and immunophenotyping of the spleen samples, although bone marrow biopsies are frequently the first and often the only sample analyzed by pathologists, since splenectomy is not clinically required in a large percentage of patients [2].

A commercially available specific marker of SMZL is lacking, and the immunophenotypical profile of SMZL is currently mainly defined on the basis on the lack of expression of markers associated with its mimickers (follicular lymphoma, mantle cell lymphoma, B-cell chronic lymphocytic leukemia/small lymphocytic lymphoma, hairy cell leukemia), including CD5, CD10, CD23, cyclin D1, CD25, CD11c, and Annexin A1 to name just a few. However, both SMZL and its mimickers can occasionally show an aberrant phenotype (e.g., expression of CD5 and/or CD23 in SMZL or lack of CD10 in follicular lymphomas (FL)), making a final diagnostic decision more difficult. The detection of the deletion of 7q might help, because it is rare in other lymphoma subtypes, while it is present in about 30–40 % of SMZL [3, 4]; its detection, however, might be impractical for routine testing and is not completely specific. So far, the only reliable marker of marginal zone differentiation is IRTA-1 [5–7], but the antibody is not commercially available. Differentiating SMZL from other lymphomas is even more problematic in bone marrow biopsies, where neoplastic cells can be scarce, morphologic criteria are less solid, the immunophenotype might differ, and genetic analysis might not work. Myeloid cell nuclear differentiation antigen (MNDA) has recently been described as a sensitive (although not completely specific) marker of nodal and splenic marginal zone lymphomas, that might be useful both in primary sites and in the bone marrow [8].

Although not widely used, TCL1A is a helpful marker in lymphoma characterization [9]. *TCL1A* (T-cell leukemia/lymphoma 1A) is a proto-oncogene member of a multigene family that includes *TCL1B* and *MTCP1* [10]. *TCL1A* maps to the 14q32.1 locus and has been shown to enhance the activity of the Akt kinase, which plays a central role in regulating cellular survival [11, 12]. In T-cell prolymphocytic leukemia, *TCL1A* acts like a classic oncogene through constitutive activation [13]. The oncogenic role of TCL1A in B-cell malignancies is less well understood: while in murine models it appears to retain a major function in the development of B-cell tumors [14], in humans, such a role is less clear.

Herling et al. reported the expression of TCL1A in a high percentage of precursor B acute lymphoblastic leukemia/lymphoblastic lymphoma, Burkitt lymphoma, and mantle cell lymphoma, while FL showed positive staining in 57 % of cases [9]. Leich et al. correlated TCL1A expression with the presence of the t(14;18) translocation in FL, with 84 % of translocation-positive cases showing TCL1A expression, while 80 % of t(14;18)-negative FL without BCL2 protein expression were also TCL1A negative [15]. These data support the hypothesis that TCL1A expression is down-regulated in B-cell tumors resembling a late B-cell program.

The present literature reports conflicting data regarding the expression of TCL1A in SMZL. Herling and co-workers [9] reported a small series of ten SMZL, which were all negative for TCL1A; variable expression of *TCL1A* mRNA was instead reported by Aggarwal et al. [16]. In a series published by Ruiz-Ballesteros et al., the protein appears to be expressed by immunohistochemistry in 80 % of cases (14/20) [17].

In this study, we analyzed the expression of TCL1A in a series of 31 consecutive cases of SMZL, which to our knowledge is the largest series so far; for 25 patients, matched bone marrow biopsies were available. Morphological, immunophenotypical, and clinical data were collected and correlated. Our data support the use of TCL1A in the diagnostic workup for SMZL.

## Methods

### Cases

Thirty-one consecutive cases of SMZL were retrieved from the archives of the Department of Pathology and Diagnostics, University of Verona. For 25 cases, one or more matched bone marrow biopsies were available.

Clinical data were available for 24 SMZL cases and included age at diagnosis, gender, spleen size, presence of autoimmune phenomena, hemoglobin, white cell count, presence of neoplastic circulating cells, presence of M component, albumin and LDH values, HBV and HCV serology, liver and nodal involvement, overall survival (OS), and disease-free survival (DFS).

Nine cases of non-Hodgkin lymphoma involving the spleen were used as controls, including five follicular lymphomas (FL), one mantle cell lymphoma (MCL), one small B-cell lymphoma/chronic lymphocytic leukemia (B-CLL/SLL), one hairy cell leukemia (HCL), and one lymphoplasmacytic lymphoma (LPL). Five normal spleen specimens (removed after traumatic injury) were also included in the study. We also added a series of lymph node cases involving lymphomas that might mimic SMZL (13 NMZL, 34 FL, 29 B-CLL/SLL, 30 MCL) and 15 bone marrow specimens involving hairy cell leukemia, which might be a differential diagnostic problem when only minimal involvement is present.

### Morphological classification

Cases were classified morphologically as described by Isaacson et al. [18]. Spleen specimens were divided into three groups according to the main pattern of neoplastic infiltration: “nodular” (white pulp effaced by tumor cells with scant red pulp infiltration), “nodular/diffuse” (marked expansion of tumor cells with variable nodular/intrasinusoidal involvement of the red pulp), and “primarily diffuse” (white pulp completely effaced with massive red pulp involvement) [18–20]. In bone marrow biopsies, the neoplastic infiltrate was defined as “sinusoidal” (rows of at least three to four cells positive for B-cell markers inside sinusoids), “interstitial” (small and discohesive groups of cells dispersed in the bone marrow interstitium), and “nodular pattern” (neoplastic cells organized in nodules that may be associated with an interstitial and/or sinusoidal component).

### Construction of tissue microarrays

Three tissue microarrays were built using a manual arrayer (Beecher Instruments). After morphological evaluation and marking of proper areas in H&E-stained slides, three 2-mm cores were taken from each block. All slides were scanned using the D-SIGHT device (Menarini Diagnostics, Florence, Italy).

### Immunohistochemistry

Immunohistochemical staining was performed using an autostainer (Leica Bond Max) using the following antibodies: CD5, CD20, Ki67, cyclin D1, CD123, TCL1, T-bet, DBA44, BCL2, BCL6, CD23, ANXA1, MNDA, and GCET1 (Table 1). MNDA and GCET1 antibodies were kindly provided by Dr. Roncador (CNIO, Madrid, Spain). Double staining was performed as previously described [21].

**Table 1:** Antibodies used in the study

Antigen	Clone	Producer	Dilution
CD5	4C7	Labvision	1:150
CD20	L26	Dako	1:250
Ki67	MM1	Novocastra	1:50
TCL1A	27D6/20	MBL	1:500
T-bet	4B10	Santa Cruz	1:100
CCND1	SP4	Neomarkers	1:10
CD123	7 G3	BD	1:100
DBA44	DBA44	Dako	1:20
BCL2	124	Dako	1:40
BCL6	LN22	Novocastra	1:10
CD23	1B12	Novocastra	1:100
ANXA1	B01P	Abnova	1:100
MNDA	235	CNIO	Pure supernatant
GCET1	RAM341	Abcam/CNIO	1:500

### Statistical analysis

Fisher exact test (one-sided and two-sided) was used for statistical analysis.

## Results

### Clinical data

Clinical data were available for 24 cases. Mean age was 62 years (range 48–79). All cases presented with splenomegaly (spleen mean main axis 22 cm, range 15–30) and all underwent splenectomy for diagnostic and therapeutic purposes. Fourteen of 24 cases (58 %) presented with liver enlargement and 17/24 with lymphadenopathy. In all patients with an available bone marrow trephine biopsy, an involvement was demonstrated.

Other relevant findings were lymphocytosis (11/24 patients, 45 %), anemia (7/24, 29 %), thrombocytopenia (14/24, 58 %), presence of serum M component (10/24, 41 %), and autoimmune phenomena (8/24, 33 %); 2/24 (8 %) patients resulted HCV positive and other two (8 %) HBV positive. One third of patients (8/24, 33 %) presented an IPI score of 3–4, while 15/24 (62 %) an IPI score of 1–2; data were not available for one case. Seven of 24 (29 %) presented LDH values above normal range; 3/24 (12 %) presented an albumin concentration below 35 g/L. Sixteen of 24 patients (66 %) had normal values of albumin, LDH, and hemoglobin; 3/24 patients (12 %) had one of these three values outside the normal range, 3/24 patients (12 %) had two abnormal values, and 2/24 (8 %) had all three values outside the reference range.

As a first line of therapy, 9/24 (37 %) patients were treated with chemotherapy, consisting of alkylating agents with or without rituximab besides splenectomy; the remaining 15 patients underwent splenectomy alone. Of these, 11 underwent chemotherapy as second-line therapy. The mean follow-up period was 69 months (range 6–233). During the follow-up period, 5/24 patients (20 %) died because of the lymphoma, after a mean period of 80 months (range 49–110); 9/24 (37 %) patients presented signs of disease progression. At the time of the last clinical observation, 7/24 patients (29 %) were free from disease after chemotherapy/splenectomy.

### Morphological classification

In the spleen, a nodular pattern of infiltration was present in 8/31 cases (26 %), a nodular/diffuse pattern in 15/31 cases (48 %), and a primarily diffuse pattern in 8/31 cases (26 %). In bone marrow biopsies, we found a mixed nodular/interstitial/sinusoidal pattern in 9/25 cases (36 %), nodular/sinusoidal in 5/25 (20 %), interstitial/sinusoidal in 4/25 (16 %), nodular/interstitial in 1/25 (4 %), purely sinusoidal in 3/25 (12 %), and purely interstitial in 3/25 (12 %) (Table 2). We detected a secretive differentiation in 4/31 cases (13 %); neoplastic involvement of the liver was documented in 13/31 cases (42 %).

**Table 2:** Synopsis of histopathological and immunohistochemical results

Case	Splenic pattern	Bone marrow pattern	CD5	CD20	Ki67	TCL1A		MNDA		T-bet	DBA44	Bcl2	Bcl6
						Spleen	BM	Spleen	BM				
2	N	NIS	−	+++	−	−	−	+	+ w	−	−	++	−
4	N	NA	−	++	−	−	NA	+	NA	−	−	+	−
14	N	NA	−	+++	−	−	NA	+	NA	−	−	++	−
28	N	IS	−	+++	−	−	−	+	+ w	−	−	+++	−
30	N	IS	−	+++	−	−	−	++	+	NA	−	++	−
1	ND	NS	−	++	−	−	−	+	+ w	−	−	−	−
6	ND	NS	−	++	−	−	−	++	+ w	−	−	+	−
11	ND	I	−	+++	++	−	−	++	+ w	−	−	+++	−
21	ND	S	−	+++	−	−	−	+	+ w	−	−	+++	+
23	ND	NIS	−	+++	−	−	−	+	+ w	++	−	+++	+
26	ND	NIS	−	+++	+	−	−	+	+ w	−	−	++	−
27	ND	NS	−	+++	−	−	−	+	+ w	−	−	+++	−
29	ND	NIS	−	+++	+	++	+	−	−	NA	−	++	−
31	D	NA	−	+++	−	++	NA	+	NA	+++	NA	++	NA
5	D	NIS	−	++	−	−	−	+	−	−	−	+	−
16	D	NS	−	+++	−	−	−	++	+ w	−	−	+++	−
17	D	S	−	+++	−	−	−	+	+ w	−	−	++	−
25	D	NA	−	++	−	−	NA	+	NA	+	−	+	−
20	D	NIS	−	+++	−	−	−	+	+ w	−	+	++	−
12	D	S	−	+++	−	−	−	+	+ w	−	++	+++	−
9	D	I	−	+++	++	++	−	+	+ w	+++	+++	++	−
15	ND	NIS	−	+++	−	−	−	+	+ w	−	+	+++	−
18	ND	NI	−	++	−	−	−	−	−	++	+	++	−
13	ND	IS	−	+++	−	++	+	+	+ w	−	+	+++	−
10	ND	NA	−	+++	−	+++	NA	+	NA	−	+	+++	−
7	ND	NA	−	+++	−	−	NA	+	NA	−	++	++	−
22	ND	I	−	+++	−	−	−	+	+	+++	++	+	+
24	ND	IS	−	+++	−	−	−	+	+ w	+++	+++	−	+
19	N	NS	−	++	−	−	−	+	+	−	+	+++	−
3	N	NIS	−	+++	−	++	+ w	+	+ w	−	+	++	−
8	N	NIS	+	+++	−	++	+	+	+ w	−	++	++	−

Cases ordered by pattern and DBA44 staining. Case numbers according to chronological order. Splenic pattern of infiltration: *N* nodular, *ND* nodular/diffuse, *D* diffuse. Bone marrow pattern of infiltration: *N* nodular, *I* interstitial, *S* sinusoidal. Immunostain evaluation, percentage of positive cells: −, <10 %; +, 11–35 %; ++, 36–75 %; +++, >75 %; *NA* not available, *w* weak, *BM* bone marrow

### Immunohistochemistry

**Fig. 1:**
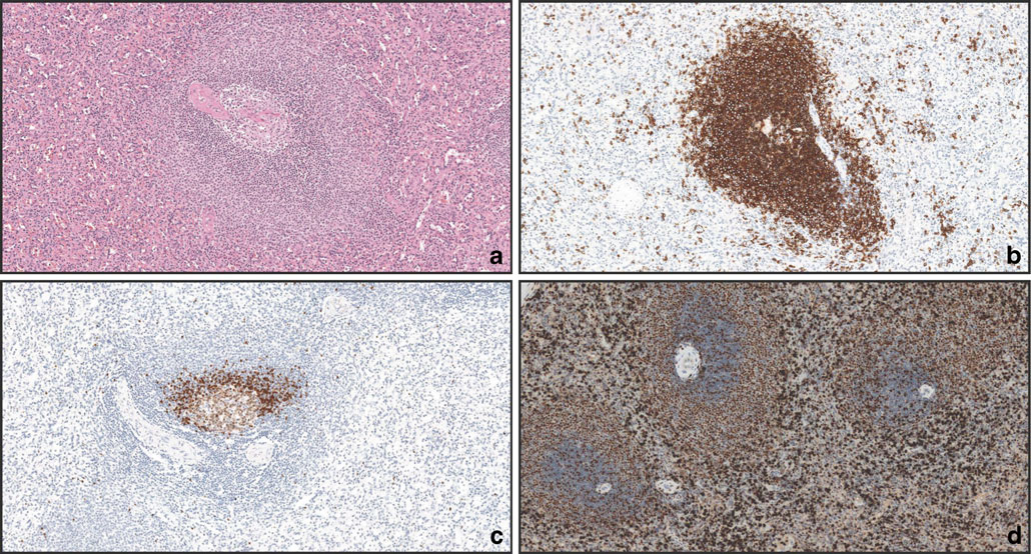
Normal spleen parenchyma. **a** Normal spleen parenchyma, H&E (×10); **b** CD20 (×10); **c** TCL1A highlights mantle cells and scattered germinal center cells but results negative in marginal zone cells (×10); **d** MNDA (×10)

Normal spleen (Fig. 1a, CD20 staining depicted in Fig. 1b) showed the typical targetoid staining for TCL1A (Fig. 1c), with germinal center cells stained at intermediate intensity, mantle cells strongly stained, and marginal zone cells unstained. MNDA, as previously described, stained both mantle and marginal zone cells (Fig. 1d).

**Fig. 2:**
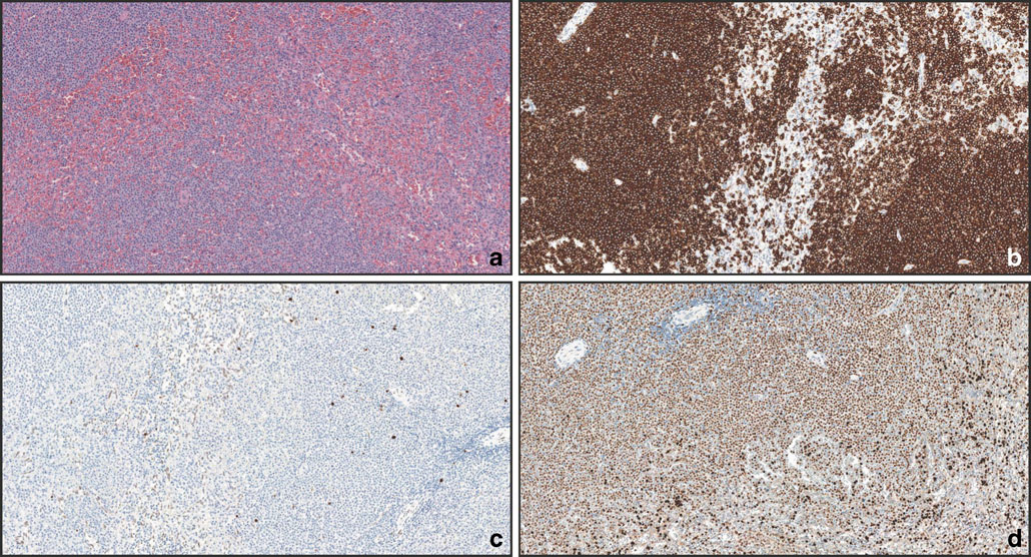
Spenic marginal zone B-cell lymphoma, nodular and diffuse pattern of infiltration. **a** H&E (×10); **b** CD20 (×10); **c** TCL1A results negative in the neoplastic cells (×10); **d** MNDA results positive in the neoplastic component (×10)

**Fig. 3:**
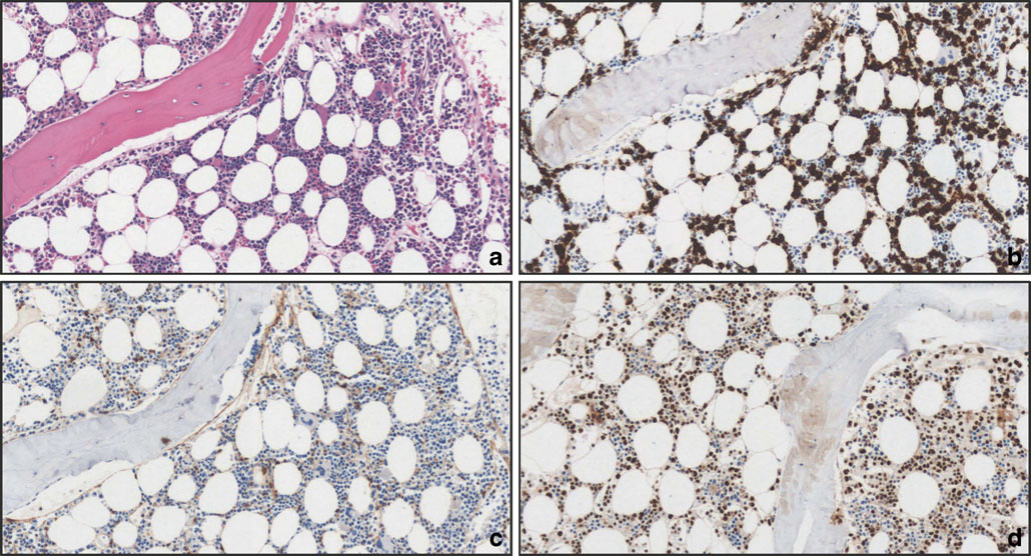
Bone marrow involvement by splenic marginal zone B-cell lymphoma showing a prevalently interstitial pattern of infiltration. **a** H&E (×20); **b** CD20 (×20); **c** TCL1A (×20); **d**: MNDA (×20)

In SMZL (Fig. 2a, CD20 staining depicted in Fig. 2b), TCL1A staining was negative in 24/31 cases (77 %) (Fig. 2c); MNDA was positive in 29/31 cases (94 %) (Fig. 2d). In the bone marrow (Fig. 3a, CD20 staining depicted in Fig. 3b), TCL1A staining was negative in 21/25 cases (84 %) (Fig. 3c); MNDA was positive in 22/25 cases (88 %) (Fig. 3d). Out of the five cases that were positive for TCL1A on spleen specimens and were paired with bone marrow biopsies, four resulted positive for TCL1A also in the bone marrow biopsy (for two cases with spleen samples bone marrow biopsies were not available). In the bone marrow, MNDA was also positive in myeloid cells, thus requiring the use of double staining for MNDA and CD20 to correctly score B cells.

**Fig. 4:**
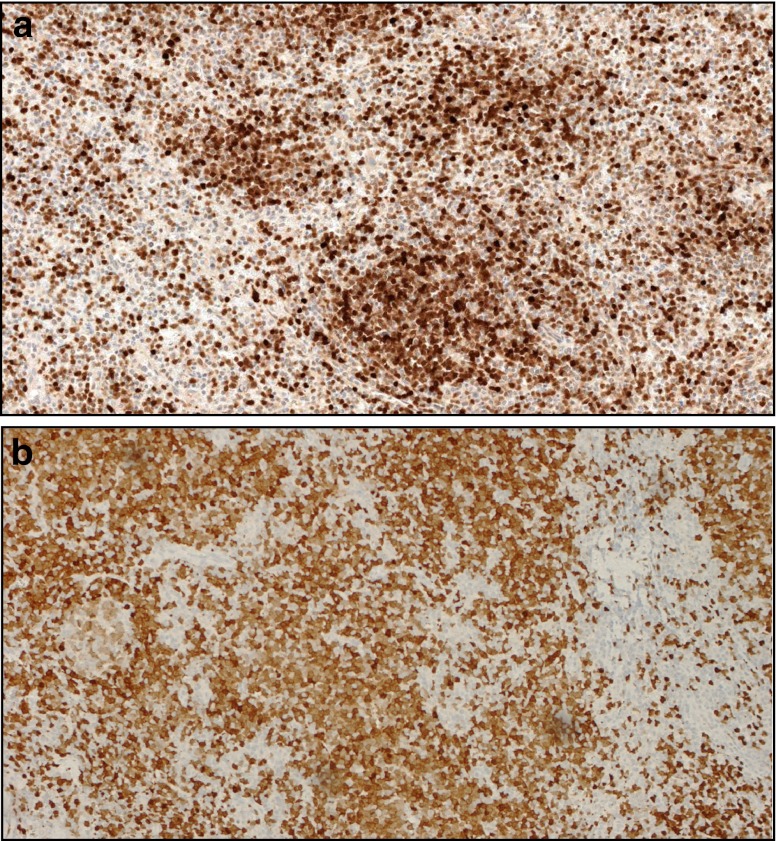
Examples of TCL1A-positive cases of splenic and nodal marginal zone lymphoma. **a** Example of a TCL1A-positive splenic marginal zone lymphoma (×10); **b** example of a TCL1A-positive nodal marginal zone lymphoma (×10); a residual non-neoplastic germinal center is also present

Thirteen of 30 (43 %) cases showed a variable degree of staining for DBA44, although only 5/30 (17 %) in more than 35 % of cells. Five of the six TCL1A-postive cases where also DBA44 positive (83 %), while 8/24 TCL1A-negative cases showed expression of DBA44 (33 %). This difference was not statistically significant using a two-sided Fisher exact test (*p* = 0.0606) but became significant when using a single-tailed test (*p* = 0.0397). T-bet resulted positive in 7/31 cases (22 %), while ANXA1, cyclin D1, and CD123 were invariably negative. In one case, the neoplastic cells were CD5 positive, but negative for CD23, cyclin D1, and TCL1A; thus, a splenic localization of CLL/SLL or MCL was reasonably excluded. TCL1A-positive cases showed no apparent peculiarities when compared to the negative ones; the staining was present usually only in part of the cells (36–75 % in six of seven cases) and with variable intensity (Fig. 4a).

Among lymph node cases involved by lymphomas that might mimic SMZL, 8/13 (61 %) of NMZL showed a positive but heterogeneous staining with a “mosaic-like” pattern (Fig. 4b). Twenty-seven of 30 MCL as well as all CLL/SLL (29/29) stained strongly and diffusely for TCL1A (three MCL cases showed a weak or heterogeneous staining that was scored as negative), while 32/34 cases of FL (94 %) were positive for TCL1A. All HCL bone marrow trephine biopsies (15/15) were positive for TCL1A. All spleen samples involved by other lymphoma/leukemia subtypes (five FL, one B-CLL, one MCL, one HCL, one LPL) stained positive for TCL1A.

### Clinicopathological correlation

No correlation was found between the type of splenic infiltration and the clinical course, including OS and DFS. We did not find any statistically significant correlation between TCL1A expression and the primarily diffuse pattern seen in 7/31 cases, nor with disease progression. In fact, we did not find any difference in terms of TCL1A expression among cases with a diffuse pattern of splenic infiltration compared to those with a nodular or nodular and diffuse pattern.

## Discussion

Due to the lack of specific markers, splenic marginal zone B-cell lymphoma is still a diagnosis of exclusion. The use of a large number of markers, even negative, allows exclusion of morphologically similar entities and thus improves diagnostic reliability. The problem is more pertinent when analyzing bone marrow specimens, since the diagnostic criteria are less reliable, and morphological evaluation is sometimes of limited help. Since only a part of the patients with SMZL undergo splenectomy before a bone marrow biopsy is taken, these specimens are often the first (and frequently the only) samples available for evaluation.

In this paper, we analyzed the expression of TCL1A in SMZL, a marker that is lost in marginal zone differentiation of normal B cells [9], the mRNA of which was detected at high levels in SMZL [22] although current literature is conflicting regarding its expression as detected by immunohistochemistry. In addition, we analyzed matched spleen and bone marrow samples and described the morphological and immunohistochemical findings in both. We also analyzed possible correlations with clinical parameters such as disease progression, albumin, hemoglobin, and LDH values.

Our series of cases showed an indolent course and good prognosis, similar to previously published studies. Only 5/24 patients (21 %) for whom clinical data were available died because of lymphoma, after a median interval of 80 months (range 49–110). Nine of 24 patients (37 %) underwent disease progression, three of whom developed transformation to diffuse large B-cell lymphoma.

Morphologically, a purely diffuse pattern was present in 8/31 (26 %) of spleen samples of SMZL, while the majority showed a classical nodular pattern with or without red pulp involvement. Three of the purely diffuse cases were also positive for DBA44 and, based on the 2008 WHO criteria, would fit in the provisional category called “splenic B-cell lymphoma, unclassifiable,” which includes a subcategory called splenic diffuse red pulp lymphoma. DBA44 was not restricted to the purely diffuse subtypes, however, since also ten other cases with mixed or nodular patterns showed a variable degree of expression (Table 2). Moreover, four cases with a purely diffuse pattern were DBA44 negative, supporting the view that this marker might not be strictly associated with a diffuse pattern of infiltration. A recent series of splenic diffuse red pulp lymphomas, however, included also cases which were DBA44 negative [23]. Interestingly, we found a higher rate of expression of DBA44 in TCL1A-positive cases, since five of the six (83 %) TCL1A-positive cases where also DBA44 positive, while 8/24 (33 %) TCL1A-negative cases showed expression of DBA44 to some extent. This association was not statistically significant using a two-tailed Fisher test (*p* = 0.0606). Since we wanted to test the positive association between TCL1A and DBA44, a one-tailed test might be appropriate which turned out to be significant (*p* = 0.0397). The small number of cases did not allow a more powerful statistical approach, but the high prevalence of double-positive cases is intriguing and warrants further investigation of the hypothesis that DBA44-positive cases might be different from the negative ones, irrespective of the growth pattern.

On the bone marrow biopsies, we confirmed what was reported so far in the literature [24] concerning the presence of a sinusoidal pattern of infiltration, which we observed (often associated with nodular and interstitial aggregates) in 80 % of cases. Regarding other immunophenotypic markers, all cases were negative for CD10, CD23, CD123, cyclin D1, and ANXA1. CD5 appeared positive in one case only, which was negative for CD23 and CCND1 excluding other entities such as MCL and B-CLL.

In our series, TCL1A was negative in almost 80 % of cases of SMZL. This finding is at variance with the high levels of mRNA of *TCL1A* detected in another series [22]. Several explanations are possible for this curious phenomenon, including low translation rate of mRNA and fast protein degradation. The reported positive staining in 80 % of SMZL [17] is more difficult to explain, also considering variations in the techniques used.

The absence of TCL1A staining seems a useful feature in defining SMZL: while in fact only 7/31 (22 %) of cases of SMZL showed positive staining for TCL1A, among cases of lymphomas that might mimic SMZL, 8/13 (61 %) of NMZL showed a mosaic-like, heterogeneously positive staining, while all nodal B-CLL/SLL (29/29) and 27/30 (90 %) nodal MCL showed strong and diffuse positivity for TCL1A (the remaining three MCL cases showed a weak or heterogeneous staining). Moreover, 32/34 nodal cases of FL (94 %) and all HCL bone marrow trephine biopsy (15/15) stained positive for TCL1A. In the spleen, five FL, one MCL, one B-CLL/SLL, one HCL, and one LPL also showed expression of TCL1A.

As previously said, among the five cases that were positive for TCL1A on spleen specimens and had paired bone marrow biopsies, one case was negative in the bone marrow biopsy. This asymmetry might be due to the interaction between neoplastic cells with the bone marrow microenvironment or simply to a lower technical performance on de-calcified samples (however, a few positive cells were always detectable).

In a study conducted in 2006 by Herling et al. [25], the expression of TCL1 has been evaluated on a series of 213 cases of CLL/SLL; in 90 % of the cases, expression of TCL1 was found by flow cytometry, immunohistochemistry, and western blotting. The highest level of expression obtained was lower than that observed in T-cell prolymphocytic leukemia (T-PLL), but correlated with expression of ZAP-70 and presence of wild-type *IGHV* genes. In splenic marginal zone B-cell lymphomas, this latter correlation has never been directly proven, but indirect evidence comes from studies that demonstrated that the presence of wild-type *IGHV* genes correlates with the presence of deletion of 7q31 [26] and that such a deletion correlates in turn with the expression of TCL1A in SMZL [17]. Further studies are therefore needed to verify this hypothesis.

In a multicenter study conducted by the Intergruppo Italiano Linfomi, Arcaini et al. [27] suggested, on a large series of consecutively diagnosed SMZL (309 cases), three parameters predictive for a shorter survival: hemoglobin less than 120 g/L, albumin less than 35 g/L, and LDH above normal values. These authors proposed a prognostic model which considers three groups: in particular, low risk (no adverse factors), intermediate risk (one adverse factor), and high risk (two or three parameters out of normal range). We did not find any correlation between the high risk group and the expression of TCL1A, although a statistically significant correlation (*p* < 0.05) is present if the three abnormal parameters are taken together and correlated with TCL1A-positive staining. TCL1A expression might therefore be a predictor of shorter survival, but the low number of TCL1A-positive cases does not allow a powerful statistical analysis.

Recently, Kanellis et al. [8] evaluated the expression of MNDA in a broad spectrum of lymphoid neoplasias, reporting that all of the 20 cases of SMZL tested were positive for this marker. In our series, we found this antigen expressed in 94 % of cases. We can confirm the usefulness of this marker, with the caveat of a strong expression in myeloid cells that might impair the recognition of the MNDA-positive lymphoid infiltrate, especially in the interstitial pattern of infiltration. In our opinion, the use of a double staining procedure, using a membrane/cytoplasmic B-cell marker such as CD20, greatly facilitates the evaluation of MNDA. Interestingly, one of the two splenic MNDA-negative cases was DBA44 positive, and the other was TCL1A positive; moreover, one case that was MNDA negative only in the bone marrow showed a diffuse pattern in the spleen. In consideration of these data and of those concerning DBA44 previously discussed, we propose to investigate the hypothesis that aberrant phenotypic/morphologic features tend to cluster together. We hypothesize that besides classical SMZL and the relatively well-described “diffuse red pulp lymphoma,” there might be a third group displaying a classical (i.e., nonpurely diffuse) pattern but showing an aberrant phenotype. The clinical and biological significance of this group, however, needs to be investigated on a larger series and possibly using high-throughput technologies to identify specific molecular signatures. In conclusion, our data support the use of TCL1A in the panel of diagnostic markers used in the differential diagnosis of splenic low-grade B-cell lymphoma; a possible biological and/or prognostic significance of this marker and the possible existence of a novel group of splenic lymphomas with an aberrant phenotype, however, need evaluation of a larger series.
